# Concussion among soccer players in the 2017 Brazilian championship – the gap between protocol and medical practice

**DOI:** 10.2217/cnc-2020-0015

**Published:** 2020-10-28

**Authors:** Cármine Porcelli Salvarani, Lucas Ribeiro de Medeiros, Fernando Henrique Sapatero, Diego Ciotta de Castro, Vinícius Simon Tomazini, Leonardo Henrique Micheletti Sotocorno, Paulo Sérgio Teixeira da Costa, Bruno Bueno Pimenta, Diego Almeida de Oliveira, Eduardo Almeida Dias, Eduardo Vinícius Colman da Silva

**Affiliations:** 1 State University of Maringá, Medicine Department – Division of Neurosurgery, Avenida Mandacaru 1590, Bloco S 05, Sala 10, Hospital Universitário, Maringá, PARANÁ, CEP 87083-240, Brazil

**Keywords:** soccer, sports-related concussion, traumatic brain injury, video analysis

## Abstract

**Background:**

The present study aims to report traumatic brain injury (TBI) among soccer players in the 2017 Brazilian Soccer Championship and discuss the protocols for concussion evaluation.

**Materials & methods:**

This is an observational study utilizing video analysis of 380 matches. TBI was considered as any event in which one or more soccer player(s) had a head trauma. For potential concussion diagnosis, we analyzed players with one of the following signs: slowness to get up, disorientation, motor incoordination, loss of consciousness, head clutching and impact seizure.

**Results:**

There were 374 TBIs in total. The average time for medical assessment was 1′35”. 13 players had concussion with an average time of 3′19″ for medical evaluation. Four players were replaced after having a concussion.

**Conclusion:**

There is a gap between concussion protocols and medical practices in Brazilian elite soccer. Further discussion about soccer replacement rules are imperative.

Sports-related concussion (SRC) is a worldwide public health concern. Concussion is a subset of mild traumatic brain injury (TBI) and it is defined as traumatically induced transient disturbance of the brain function that involves a complex pathophysiological process. Based on acute injury characteristics, concussion is placed at the less severe end of the brain injury spectrum [1].

In the world of sports, the highest incidence of SRC occurs in American Football, hockey, rugby, soccer and basketball. In soccer, head injury can be the result of a collision between two heads (or other body parts), or between a head and the ground, goal post, other unknown object or an unexpected ball hit. Due to the global popularity of soccer, the overall contribution of this sport to the total SRC amount is likely to be significantly more than other sports [2].

According to recent publications, the neurological understanding of SRC consequences and its effect on the lives of athletes, especially regarding soccer, is still not complete. As a result, the development of effective tools for proper management of these injuries has been reported by current research. The sideline assessment of SRC in soccer is challenging due to the variability of its clinical presentation, the specific environment, the reliance on athlete-reported symptoms and the varying specificity and sensitivity values of sideline assessment tools. The correct assessment of suspected concussion immediately after injury is an important practice for early diagnosis, for appropriate management of athletes with TBI and for determining whether it is safe for a player to return to the match [3,4].

There are protocols provided by the consecutive 3rd, 4th and 5th International Conferences on Concussion in Sports that clinicians are recommended to follow when an athlete shows any sign of concussion [5–7]. The latest Sport Concussion Assessment Tool (SCAT), developed by Consensus Statement on Concussion in Sports, was adopted by Fédération Internationale de Football Association (FIFA; Zürich, Switzerland) [8,9]. However, previous reports using video analysis from the 2014 Men’s FIFA World Cup and the 2016 Men’s Union of European Football Association (UEFA) found that many potential concussion events were not correctly evaluated, showing a lack of congruence between these protocols and practices on field [10,11].

This study is the first specific report with video analysis of TBI among soccer players in the Brazilian Series A Soccer Championship, regarding the complete epidemiology, undertaken with an aim to verify the characteristics of TBI in Brazilian Elite soccer and to discuss the medical protocols for concussion in soccer matches.

## Materials & methods

This is an observational study concerning video analysis of all 380 matches (38 rounds, 10 matches each round) of the 2017, Series A, Brazilian Soccer Championship, based on recent publications related to SRC with video analysis [10–14]. The videos of players with suspected TBI were first analyzed at the same time the matches took place by a team of ten trained medical students of the State University of Maringá (Paraná, Brazil), under the guidance of a neurosurgery professor. After first analysis, all suspected TBIs were reviewed by the research coordinator.

### Guideline & coding of events

**Figure 1.**
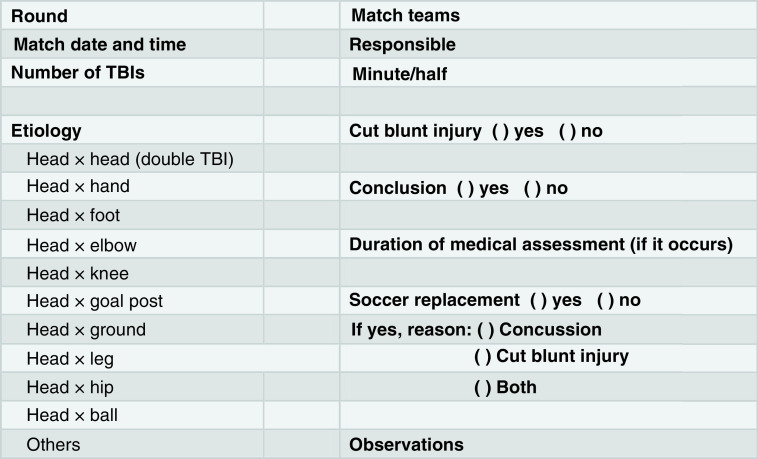
Protocol for collection of data for traumatic brain injury.

A guideline was built to record data about TBI characteristics (Figure 1). In order to fulfill the guideline, TBI was considered as any event in which one or more soccer players had a head trauma (head × head, head × unexpected ball hit or head × elbow, for instances). On multiple occasions, a Brazilian soccer player claimed to have a TBI, but they were pretending to gain an advantage in the match. To minimize this situation, all videos with suspected TBI were inserted in a smartphone application platform (WhatsApp^®^, Facebook, CA, USA), shared among all students and checked by the research coordinator. In addition to the videos, the television match commentary was taken to add some information about TBI. A ‘head-to-head’ TBI was considered a double TBI, meaning two TBIs occurring at the same moment.

While we acknowledge that concussions can also occur from contact to other parts of the body, besides the head and neck area, this study limited the assessment of concussions only after direct trauma with the head area. A potential concussion diagnosis was defined as any event of TBI (through direct contact among athletes, unexpected ball hit or object in the environment) that left the soccer player unable to immediately resume play following impact. Further, for concussion diagnosis, we analyzed players with one of these visual signs: slowness to get up, disorientation, motor incoordination, loss of consciousness, head clutching and impact seizure. When a soccer player was replaced due to TBI, the after-match medical information was taken from media information and/or from the official soccer team medical statement.

### Medical assessment

The length of sideline medical assessment was systematically recorded in all cases of TBI that required a medical attendance. The start of medical assessment was considered to be when medical staff reached the player, whether on the field or on the sidelines. The end of the medical assessment was established by the medical decision of the player returning to play or being replaced.

### Patient & public involvement

Patients and/or public were not involved in this research.

## Results

Across the 380 matches a total of 374 TBIs occurred, averaging almost one TBI per match (0.98). There were 30 cut blunt injuries among TBIs and three players were replaced to have wounds sutured due to the extension of their injuries. The other 27 players were treated by compressive dressing with swimming caps and were not replaced. The overall findings, with etiology, cut blunt injuries, concussion and players’ replacements are shown on Table 1.

**Table 1.** Characteristics of traumatic brain injury concerning overall events, relation with matches, etiology, cut blunt injury, concussion and players’ replacements (total number of matches: 380, total number of traumatic brain injury: 374).

TBI per match	0.98	Matches with TBI	195
TBI per round	9.98	Matches without TBI	185
**Etiology**		**Cut blunt wound**	30
– head × head (double TBI)	91 (182)	– head × head	19
– head × elbow	86	– head × elbow	6
– head × foot	29	– head × foot	4
– head × unexpected ball hit	17	– head × knee	1
– head × knee	12		
– head × leg	11	Player replacement after TBI	12
– head × shoulder	10		
– head × hip	9	Concussion (3.5% of all TBI)	13
– head × hand	7		
– head × ground	4	Player replacement after concussion	4
– head × others	7		^1^

TBI: Traumatic brain injury.

**Figure 2.**
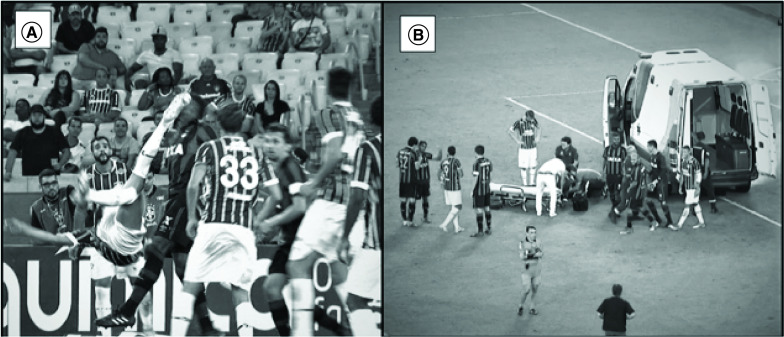
Player with concussion after head x foot trauma. **(A)** Player’s photo showing the moment of traumatic brain injury with later impact seizure. **(B)** Ambulance on the field during medical assessment to remove the player to a hospital.

Thirteen players had concussion (3.5% of all TBIs) and only four of them were replaced. Of these players who had concussion, only two were assisted by an Ambulance in the field. These two players had impact seizure and, in both situations, were removed to a hospital (Figure 2). The post match computed tomography and MRI of both players were normal.

The maximum number of TBIs in one match was seven, but no players were replaced in that match. Three of those TBIs occurred in just one play that involved three players (a triple head TBI).

There were 82 events of TBI where medical assessment was not required. In general, the mean time for medical assessment among the remaining 292 TBIs was 1′35” (from 48” to 7′40”). Considering the 13 players who had concussion and the 12 players who were replaced (four due to concussion, four due to head cut blunt injury, two due to dizziness, two due headache), the mean time for medical evaluation was 3′26” and 3′19”, respectively (see Tables 2 & 3). If the two reported severe TBIs, for whom an ambulance was required on field, were excluded from the average, the mean time for medical evaluation goes down to 2′43” for players with concussion and 2′54” for replaced players after TBI. In both situations, the length of medical evaluation was very similar despite the occurrence of concussion. Comparing the overall mean time for medical assessment and players with concussion, there was an increase of 1′51”.

**Table 2.** Relationship between etiology of concussion, player replacements and length of medical assessment after traumatic brain injury (number of concussions: 13; players replacement after concussion: 4).

Etiology	Number of concussion	Player replacement after concussion	Time for medical evaluation (mean time: 3′26”/2′43”^†^)	Observations
Unexpected ball hit × head	4	0	Case 1: 2′40” Case 2: 2′30” Case 3: 2′50” Case 4: 2′42”	
Elbow × head	3	1	Case 5: 2′30” Case 6: 2′45” Case 7: 2′50”	Maintained disorientation
Foot × head	2	2	Case 8: 2′50” Case 9: 7′40”^‡^	Cut blunt injury associated Player removed to hospital
Knee × head	2	1	Case 10: 7′10”^‡^ Case 11: 2′45”	Player removed to hospital
Head × head	1	0	Case 12: 2′50”	
Hip × head	1	0	Case 13: 2′40”	

† Mean time for medical evaluation without cases 9 and 10.

‡ In both situations, players were removed from field by ambulance to hospital.

**Table 3.** Relationship between etiology, causes of player replacements (concussion and non-concussion events) and length of medical assessment after traumatic brain injury (number of replacements: 12).

Etiology	n	Cause/observation	Time for medical evaluation (mean time: 3′19”/2′54”^†^)
Knee × head	1	Case 1: impact seizure/player removed to hospital	7′10”^‡^
Foot × head	2	Case 2: impact seizure/player remove to hospital Case 3: concussion/cut blunt injury associated	7′40”^‡^ 2′50”
Head × head	4	Case 4: subgaleal hematoma and dizziness Case 5: cut blunt injury Case 6: dizziness Case 7: cut blunt injury	2′00” 5′30” 5′00” 2′50”
Elbow × head	4	Case 8: concussion/maintained disorientation Case 9: cut blunt injury Case 10: cut blunt injury Case 11: headache	2′30” 1′45” 1′40” 2′20”
Shoulder × head	1	Case 12: headache	2′40”

† Mean time for medical evaluation without cases 9 and 10.

‡ In both situations, players were removed from field by intensive care unit ambulance to hospital.

The main characteristics of players with concussion and replaced players after TBI are described on Tables 2 & 3.

## Discussion

In the history of soccer, there have been reports of some unforgettable TBI occurrences: Leonardo and Tab Ramos, in a match between Brazil and USA at the 1994 FIFA World Cup; Petr Cech, goalkeeper of Chelsea, during a match against Reading in the 2006 Premier League Championship; and Fernando Torres at the La Liga Championship in 2017. In Brazil, a soccer player called Vagner Bacharel died 6 days after a TBI during a match in 1990. The misdiagnosed acute epidural hematoma was the etiology of his death. Year after year, TBI among soccer players has been growing and the concern with their assessment has been reported [15–18].

Video analysis does not provide full information about TBI and medical assessment; however, despite its limitations, it may be a useful adjunct to the sideline assessment of a possible concussion [12]. In the National Football League, a very recent study showed that visual signs for potential concussion detected by video analysis cannot be made on-field alone. The video analysis is a part of the comprehensive sideline/acute evaluation of concussion and the diagnosis remains a multimodal clinical decision [19].

Recent studies utilized video analysis at the 2014 Men’s FIFA World Cup and the 2016 UEFA European Championship [10,11]. According to these publications, this first study among soccer players in Brazil, supported by video analysis, showed how the concussion was evaluated on sideline. In addition, it was studied the overall epidemiologic data of TBI, the length of medical assessment of players who had concussion and/or were replaced after TBI.

In terms of soccer biomechanics, head-to-head impacts and unexpected ball hits with great speed each result in the greatest form of head acceleration and, consequently, come with higher risks of concussion [2,20]. This is important because, in our findings, the most frequent TBI event was head-to-head (91 events resulting in 182 TBIs); however, there were four concussions after an unexpected ball hit (out of 17 events) and only one case of concussion after head-to-head impact.

Updated protocols for sideline assessment of an athlete with a concussion were published following each of the International Conferences on Concussion in Sport in 2008, 2012 and 2016 [5–7]. The Concussion in Sports Group recommendations assert that when an athlete shows any features of a concussion, the athlete should be evaluated by a physician or another licensed healthcare provider onsite, assessed using SCAT or other sideline concussion assessment tools and prevented from return to play in the event of a positive diagnosis. The SCAT-5 is the assessment tool supported and endorsed by FIFA [9].

In the Men’s 2014 FIFA World Cup and 2016 UEFA European Championship, 63 and 72.5% of athletes who experienced a potential concussion event were not medically assessed, respectively. All players with a potential concussion event that were medically assessed resulted in no replacement after TBI [10,11].

In this study, only four players out of 13 with a concussion during the Championship were replaced on the pitch. This included two players who were assisted by an ambulance on the field and removed directly to the hospital due to an impact seizure and one player who had an extensive cut blunt injury associated with the concussion. Just one player was replaced after the concussion without any associated injuries. Besides, on two different occasions, video images showed that the decision to return to the match was motivated and pressured more by the athlete’s desire than the medical staff itself.

Soccer officials should be trained to recognize signs and symptoms of concussion in order to know when to ask for a medical assessment [21]. After that, physicians must provide care along the continuum of the concussion, from the acute injury to return-to-play decisions [2]. According to SCAT-5, which has been adopted by FIFA [9], the protocols cannot be performed in less than 10 min. However, during the 2014 FIFA World Cup Championship, the mean time for player assessment after suspected concussion was 1′47″ (range: 1′04″ to 3′) [11].

In our findings, in 374 occasions of TBI, 82 players did not receive medical attendance. The mean time for the 292 players with TBIs who did receive assessment was 1′35″ (range: 48″ to 7′40″). Considering only the 13 players who had a concussion and the 12 players who were replaced, the mean time for medical evaluation was 3′26” and 3′19”, respectively. If the two reported severe TBIs, which required an ambulance was on field, were excluded from the average, the mean time for medical evaluation goes down to 2′43” for players with concussion and 2′54” for replaced players after TBI. In comparison with the 2014 FIFA World Cup, there was an increased amount of time given for player assessment in the 2017 Brazilian Soccer Championship.

In November 2018, the Brazilian Soccer Confederation (CBF; Rio de Janeiro, Brazil) organized a Medical Symposium at Rio de Janeiro and discussed the protocols for concussion evaluation in Brazil. At this Symposium, the idea of a video review for concussion evaluation and an extra soccer player replacement were discussed [21]. Following this Symposium, the CBF started the discussion about medical training and protocols for concussion assessment, but no formal protocol for concussion assessment has been endorsed by CBF up to now [22].

The North American soccer league Major League Soccer implemented programs with video review and league spotters to provide medical staff detailed early detection of concussion. Major League Soccer data suggest that the video is effective in increasing knowledge on concussion. Major League Soccer, US Soccer and the National Women's Soccer League jointly hosted a conference on April 2017 in New York and discussed the temporary replacement of players with suspected concussion to allow time for adequate medical assessment [23].

Many sports, including basketball, rugby, American Football and ice hockey, have formal and feasible protocols for concussion. According to their rules, the athletes can be temporarily replaced to be evaluated on sideline and, after protocol tests, they can return to play or not.

Despite standardized protocols for concussion in soccer that have been endorsed by FIFA, the main concern that arises from recent studies, including this one, is that there is a gap between these formal protocols and the sideline medical assessment in practice. In addition, the recognition of injury and assessment always occur in a time-pressured environment, requiring rapid disposition and decision-making [5]. There is no doubt that the duration of this evaluation is very far from the ideal in order to preserve the player’s integrity.

## Conclusion

Soccer rules forbid temporary replacement of players during a match. This fact is potentially the biggest challenge in this kind of sport on how to deal with concussion assessment when compared with other team sports. It means that, in the soccer environment, the medical professionals are pressured to perform concussion protocols on the sideline as fast as possible because of a huge team disadvantage in the dynamic of the game without one player on the field. Our results among Elite Soccer Players in Brazil confirm that the length of medical evaluation on the sideline is not long enough for an adequate performance. Consequently, the concussion and other potential neurologic injuries may be misdiagnosed, with a life-threatening risk to the professional soccer player.

## Future perspective

Considering the previous studies already mentioned, this report suggests an alternative to the challenging issue of soccer’s replacement rules. This study supports the idea of creating a temporary replacement. Once a player is diagnosed with a potential concussion, the player with a concussion should be temporarily replaced, maintaining a team with all players on the field while the player is assessed. They can then return to play following a full medical evaluation, if it is medically advisable. This will provide time for an adequate sideline evaluation and preserve the integrity of soccer players, respecting the dynamic and the environment of the sport.

**Executive summary**

- Despite the limitations of video analysis, it may be a useful tool for recognizing possible concussion and it may be a useful adjunct to the sideline medical assessment.
- Traumatic brain injury is more often than expected and ‘head-to-head’ event is the majority of related head injury (48,6%) among soccer players in 2017 Brazilian Championship.
- Among 13 players who had concussion, the unexpected ball hit was the main cause (four cases) and only four players were replaced after concussion, two of these were removed directly to hospital after impact seizure.
- The mean time for medical evaluation of the 13 players with concussion was 3′26”. Despite standardized protocols for concussion in soccer endorsed by FIFA, the main concern that arises from this study is that there is a gap between these formal protocols and the sideline medical assessment in practice on Elite Soccer in Brazil.
- In the soccer environment, medical professionals are pressured to perform concussion protocols on the sideline as fast as possible because of a huge team disadvantage in the dynamic of the game without one player on the field. Our results confirm that the length of medical evaluation on the sideline is not enough for an adequate performance. Consequently, the concussion and other potential neurologic injuries may be misdiagnosed, with a life-threatening risk to the professional soccer player.
- This report supports the idea of creating a temporary replacement in soccer’s replacement rules. This will allow time for an adequate sideline concussion evaluation and preserve the integrity of soccer players, respecting the dynamic and the environment of the sport.
